# Management of Lumbosacral Myelomeningocele

**Published:** 2016-12-27

**Authors:** Peter M. DeJong, Nicholas S. Adams, Robert J. Mann, John W. Polley, John A. Girotto

**Affiliations:** ^a^Michigan State University College of Human Medicine, Grand Rapids, Mich; ^b^Plastic and Reconstructive Surgery Residency, Grand Rapids Medical Education Partners, Grand Rapids, Mich; ^c^Pediatric Plastic and Craniofacial Surgery, Helen DeVos Children's Hospital, Grand Rapids, Mich

**Keywords:** myelomeningocele, spina bifida, paraspinal fascial turnover flap, neural tube defect, fascial flap

## DESCRIPTION

**Figure 1:**
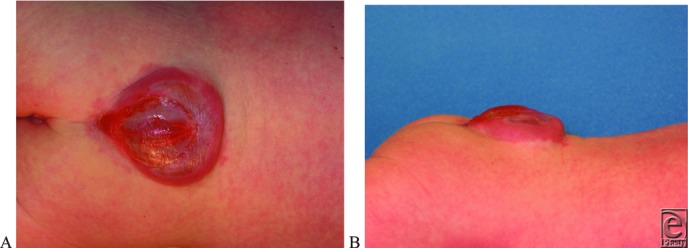
(a, b) Preoperative posterior and lateral photographs demonstrating the lumbosacral myelomeningocele prior to neurosurgical repair and soft-tissue coverage.

A newborn female patient presented with a prenatally diagnosed lumbosacral myelomeningocele (MMC) (Figs 1*a* and 1*b*). Four hours after cesarean delivery, the child was taken for neurosurgical repair. Plastic Surgery was consulted for soft-tissue closure.

## QUESTIONS

1. **Discuss the embryology of neural tube closure.**
2. **What are neural tube defects (NTDs)?**
3. **What are the risk factors for developing NTDs?**
4. **What techniques have been described for soft-tissue coverage of MMC?**

## DISCUSSION

**Figure 2:**
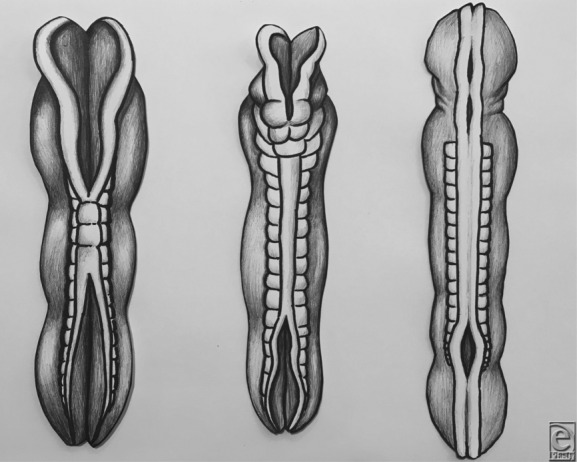
Neural tube closure. Neural tube closure progressing in a zipper-like fashion starting centrally and moving cranial and caudal (left and center). Failure of caudal neuropore closure results in myelomeningocele (right).

Neural tube closure occurs early in human development as part of primary neurulation. It is completed between the third and fourth weeks of embryonic development. The neural plate, derived from neuroectodermal cells or neural crest cells, forms the neural groove during week 3. Progressive bending of the groove leads to the eventual fusion of the resulting neural folds. Beginning at the level of the fifth somite, near the future convergence of the spinal cord and hindbrain, the resultant cranial and caudal neuropores close bidirectionally in a zipper-like fashion (Fig 2). Cranial neuropore closure is completed by day 25, whereas the caudal neuropore closes on day 28.^1^ Successful completion of this closure requires the precise coordination of cellular genetic regulation, molecular processes, and the physical forces they generate.^2^

NTDs arise from failed migration of neural crest cells. Because the cranial and caudal ends of the neural tube are the last to close, disruptions in neural crest cell migration will lead to varying degrees and varying locations of defects. Failure of cranial closure will lead to exencephaly/anencephaly, a fatal condition.^2^ Failure of caudal neuropore closure leads to open defects such as meningocele and MMC or spina bifida cystica (Fig 2). MMC is the most common NTD that is compatible with life, with an incidence of 0.44 to 1 per 1000 live births. Following the failed closure in MMC, agenesis of fetal vertebral arches leads to a protrusion of spinal cord, nerve roots, and meninges in a sac that protrudes through the skin of the lumbar area (Figs 1*a* and 1*b*). In surviving individuals with MMC, disruption of the cord at the affected area leads to a lifetime of facing incontinence, numbness, and abnormalities of the lower extremities. The exposed nature of the sac leads to greatly increased risk of infection in the neonatal period.^3^

The factors affecting neural tube closure are varied and complex. Genetic influences during early fetal development play an integral part in neural migration, especially epigenetic regulation through DNA methylation and histone modifications. Some trisomies (3, 8, X) are linked to NTDs, without specific causative genetic links identified yet.^4^ Environmental factors are also commonly cited as modifiers of these epigenetic processes and the transcriptional processes they drive. Folate antagonist medications such as methotrexate and valproic acid increase rates of NTDs 6-fold.^2^,^5^ Folic acid supplementation has been linked to significantly decreased NTD occurrence. Maternal obesity and diabetes have also been implicated as risk factors for NTDs due to alterations in genes associated with glucose metabolism.^2^ Interestingly, factors such as smoking and drug use have not shown significant association.^5^

**Figure 3:**
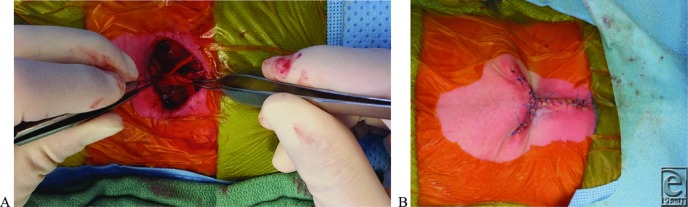
(a, b) Intraoperative paraspinal turnover flap technique and skin closure. The fascial closure technique (a) adds structural support to the neurosurgical repair, decreasing the risk of cerebrospinal fluid leak and infection. Three-sided skin closure (b) was formed without removal of redundant tissue.

**Figure 4:**
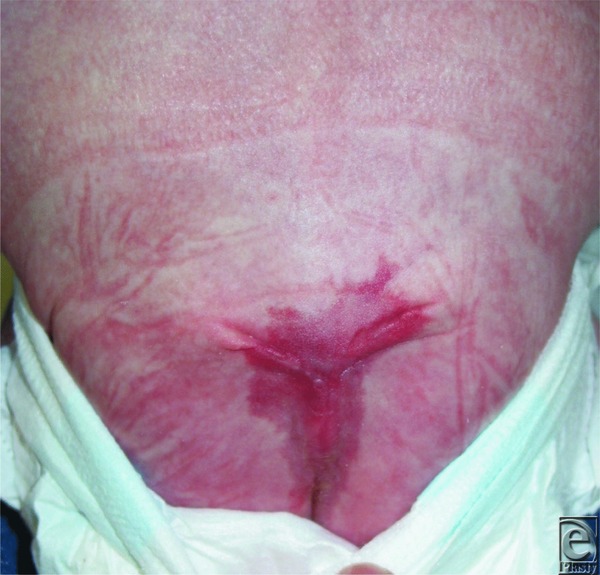
Postoperative photograph taken 2 months following closure.

Soft-tissue coverage provides structural support to the neurosurgical dural repair, which helps avoid cerebrospinal fluid (CSF) leaks. Furthermore, the barrier provided by the soft-tissue flap separates the CSF from the skin, a potential source of meningitis. There are many options for soft-tissue coverage following neurosurgical repair of the MMC. Defects that are too large to be closed by primary closure can be achieved by myocutaneous or fasciocutaneous flaps. Myocutaneous closure has been shown efficacious for repair, utilizing latissimus, gluteal, and paraspinous muscles.^6^ These muscle flaps are able to fill dead space in larger defects with robust, vascularized tissue. Fascial turnover flap closure using bilateral paraspinous muscle fascia is also widely used and has been shown to provide a strong repair that is well vascularized (Figs 3*a*, 3*b*, and 4). These are effective when the defect is caudal to the paraspinous muscles. Paraspinous techniques have also been shown to reduce cord tethering as a complication following repair.^7^
